# Integrative Health and Wellness Coaching in Occupational Therapy—A Scoping Review

**DOI:** 10.1155/oti/5937689

**Published:** 2026-04-23

**Authors:** Alyssa M. Smith, Kelli Kauffroath, Karen Westervelt, Victoria Priganc

**Affiliations:** ^1^ Department of Rehabilitation and Movement Sciences, University of Vermont, Burlington, Vermont, USA, uvm.edu; ^2^ Osher Center for Integrative Health, University of Vermont, Burlington, Vermont, USA, uvm.edu; ^3^ Dana Health Sciences Library, University of Vermont, Burlington, Vermont, USA, uvm.edu

## Abstract

**Background:**

Integrative health and wellness coaching (HWC) is an emerging evidence‐based profession that promotes healthy behavioral change to reduce lifestyle disease risk factors. This scoping review identifies parallels between occupational therapy and HWC.

**Purpose:**

The purpose of this study is to examine the following question: Are HWC practices already being implemented in occupational therapy, and if so, how?

**Data Sources:**

Searches were conducted for peer‐reviewed articles published from 2013 to 2025.

**Study Selection and Data Collection:**

Inclusion criteria included articles published in English and the use of health promotion interventions by occupational therapists or an interprofessional team of healthcare providers including occupational therapists.

**Findings:**

Twenty‐five articles fit the inclusion criteria. This includes qualitative and quantitative studies highlighting occupational therapists using HWC skills and strategies within their health promotion interventions. Multiple lifestyle disease risk factors were targeted through healthy behavior changes including increasing physical exercise, education regarding nutrition, and goal setting. These health‐promoting interventions were provided across a spectrum of diagnoses and clinical settings.

**Conclusions:**

Results demonstrated a connection between HWC and the holistic and health‐promoting practices of occupational therapy. This review adds important evidence to the literature supporting the use of HWC skills in occupational therapy practice.

**Summary**

In this scoping review, the authors looked at how health and wellness coaching (HWC) practices are similar to what occupational therapists already do. They looked to see if occupational therapists were using HWC techniques and how they were using them. They found 25 articles that talked about this topic in occupational therapy practice. These articles showed that occupational therapists often use HWC skills and strategies when helping people with different health issues. They focus on things like setting goals to improve health during their sessions. This happens in various healthcare settings and with different types of patients. Overall, the review shows that there is a natural connection between what occupational therapists already do and the practices of HWC. This suggests that HWC techniques could be more formally used in occupational therapy to improve patient care.

**Key Points for Occupational Therapy**

This article highlights:

• The connection between HWC and occupational therapy practice to improve clinical practice on a local and global scale.
• The adaptability of occupational therapy as a profession to adopt novel clinical interventions to implement in practice.
• An opportunity to expand the scope of occupational therapy future education and policy in the field.

## 1. Introduction

Recently, a call to action has been made to highlight the occupational therapist′s ability to not only treat the impact of lifestyle and chronic diseases but to engage in health‐promoting interventions aimed at preventative strategies before the disease begins to affect daily life [1–3]. The scope of occupational therapy practice is known for its breadth in treatment and impact on functioning across the lifespan; it has now become more evident to researchers and clinicians alike of its potential to prevent the consequences of lifestyle disease through health and wellness promotion to improve client quality of life comprehensively ([1]; [4]). Per the World Health Organization (1998), health promotion is defined as a process of enabling people to increase control over, and to improve, their health. As further research defines the risk behaviors of chronic diseases and their impacts on population health, the occupational therapy profession is poised to be a part of the preventative medicine team prepared to address the newest pandemic of lifestyle diseases.

According to the National Center for Chronic Disease Prevention and Health Promotion (NCCDPHP) (2023), 6 in 10 adults have a chronic disease and 4 in 10 have two or more. Many of these noncommunicable diseases have preventable factors that can reduce risk or control long‐term symptoms called lifestyle diseases [5, 6]. Lifestyle factors can include tobacco use, aerobic exercise engagement, and nutrition intake. Such lifestyle factors can be contributing factors to population health issues such as obesity, hypertension, and dyslipidemia [6–8]. One intervention that has been identified as an effective strategy in clinical populations to address the treatment of lifestyle diseases is HWC [9–11]. Coaching as a concept has been present in occupational therapy practice since 2007 when it was identified as an enabling skill for practice, increasing its popularity in use [12]. However, a scoping review conducted by Graham et al. [13] noted that although coaching has been a prominent feature in occupational therapy intervention since its highlight in the literature, it has lacked a clear theoretical underpinning guiding its practice. In contrast, per a systematic review that developed a compendium of HWC literature, a common definition of HWC was developed: A client‐centered process that assumes a working relationship that develops between a client and clinician to advance healthy lifestyle behavior change with tools such as goal setting, nonjudgmental dialogue, and accountability [10].

More specifically, HWC encompasses partnering with clients to elicit and sustain healthy behavior change through a variety of strategies focused on client autonomy and using components of established theoretical frameworks such as motivational interviewing [14], positive psychology [15], social cognitive theory [16], and the transtheoretical model of behavior change [17]. This client‐led approach uses these theoretical tenets to help clients discover what health‐related behavioral changes they are ready to make and assist them with making self‐identified goals. Additionally, in HWC, the clinician is not considered the expert in anything other than the behavioral change process. This improves client autonomy, self‐reflection, and focuses on the client identifying the psychosocial factors that contribute to their motivation to initiate and maintain behavior change (Kreisberg and Marra 2017; [18]). In this context, the healthcare provider identifies as the coach to elicit this working partnership and takes into account the client or patient′s needs, personal goals, and lifestyle factors [18, 19]. These tenets of HWC bear many similarities to the field of occupational therapy but are still distinct from its clinical practice, which leans toward a more reactive approach to chronic disease in the current healthcare landscape.

**Figure 1:**
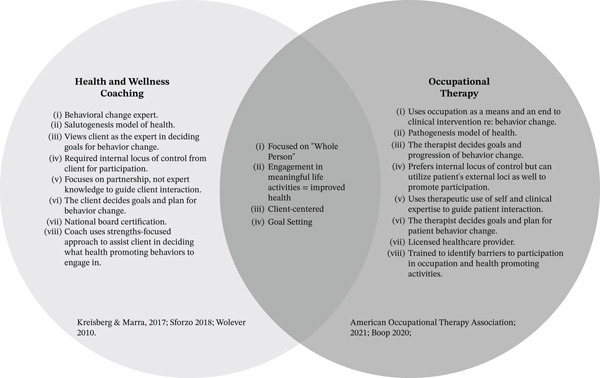
Comparison of HWC and occupational therapy.

Several aspects of HWC mirror the biopsychosocial approach of occupational therapy [20]. HWC facilitates health‐related behavior changes through a client‐centered approach that focuses on encompassing many areas of life including the physical and social environment, personal and professional development, and spirituality. These areas are in addition to physical movement and healthy nutritional intake, which are the more traditional areas of focus for healthy behavior change [21, 22]. This integrative approach of addressing the whole person aligns with the Occupational Therapy Practice Framework: Domain and Process [23] as its framework states that occupational therapists can elicit health, well‐being, and participation in life through engagement in occupation. HWC also demonstrates a client‐centered nature, focusing on all aspects of one′s functioning and environment to improve wellness. The occupational therapy profession embraces the belief that what people can do, be, and strive to become is the crux of who they are, and if addressed well, health and well‐being are by‐products [23]. See Figure 1 for a deeper comparison of the foundational constructions of the two professions. Given these similar conceptual underpinnings between these two integrative professions, a research question was developed to understand the relationship between HWC and occupational therapy: Are HWC practices being implemented in occupational therapy patient care, and if so, how? Understanding its current utilization in the occupational therapy profession, HWC as a formal clinical strategy has the potential to be a primary preventative strategy against the effects and development of lifestyle disease in clinical populations.

Evidence of other allied health professions utilizing HWC is abundant in the literature. Physical therapists, nurses, medical assistants, and even pharmacists have been using HWC skills in their treatment plans [11, 24–26]. Literature in the field of occupational therapy describing HWC methods, however, is minimal. Yet, there is a wealth of literature describing occupational therapy as a well‐equipped field to engage in health promotion and wellness strategies with clients and take on a preventative role in health care by focusing on well‐being and healthy behavior change [1, 27, 28].

Currently, no literature defines the need for, nor the implementation of, a formal integrative HWC intervention in occupational therapy practice. This paper is aimed at examining how health‐coaching skills are currently utilized within occupational therapy literature to explore the potential for HWC as a formal intervention strategy in practice. A scoping review was conducted to map the occupational therapist′s role in health and wellness promotion and characterize the implementation of HWC skills. This process provides a basis for evaluating HWC as a preventative intervention in community and clinical settings to support health‐related outcomes.

## 2. Methods

### 2.1. Positionality Statement

Two authors (A.M.S. and K.W.) have extensive experience in health coaching (1+ and 5+ years). The first author is both an occupational therapist and health coach with direct experience providing coaching skills in the clinical field. KW also has experience developing health coaching curricula. Additionally, VP is an occupational therapist with a background in developing occupational therapy curricula and works in the academic field of occupational therapy. KK is a health sciences librarian with a rigorous background in literature‐searching methods.

### 2.2. Approach

This scoping review was structured using the Arksey and O′Malley [29] framework, with the objective of identifying and mapping current published research on the incorporation of HWC into the health promotion practices of occupational therapists. However, in recognition that this seminal work does not reflect current database and searching capabilities, a protocol was drafted utilizing the Joanna Briggs Institute (JBI) methodological approach for scoping reviews (2014) and reported in accordance with the PRISMA Extension for Scoping Reviews (PRISMA‐ScR) checklist. The review protocol was prospectively registered with the Open Science Framework (OSF) on May 30, 2023 (10.17605/OSF.IO/XPE95), where supplemental research artifacts, including full search strategies, data extraction instruments, and screening logs, are hosted [30]; [31–33]).

### 2.3. Eligibility

Eligible articles were limited to English‐language, peer‐reviewed empirical research, completed case studies, and trials published between 2013 and 2025 that reported at least one qualitative or quantitative outcome. Conversely, studies were excluded if they consisted of gray literature, “call to action” papers, or theoretical frameworks lacking participant trials, as well as any publications where an occupational therapist was not involved in the intervention or analysis. Systematic or scoping reviews were used only for backward citation searching and were not included in the analysis. A 10‐year publication window prior to the study′s inception in 2023 was established to ensure the capture of contemporary evidence and modern clinical frameworks within occupational therapy; the window was subsequently extended to December 2025 via a “date added” search rerun to maintain currency during the peer‐review process.

### 2.4. Study Identification and Selection

The researchers collaborated with a university health sciences librarian to develop a methodological approach to identify and retrieve relevant published primary studies highlighting occupational therapists engaging in health promoting interventions. The Arksey and O′Malley Framework [29] was used with updated enhancements to search rigor informed by expectations laid out in the JBI Scoping Review Protocol template [32]; this approach was applied in tandem with the project′s PCC scoping question (participant, concept, and context) and a priori protocol eligibility criteria. The multiphased strategy involved constructing and executing searches in the selected databases identified in the study protocol, manual screening of included publications′ reference lists and key occupational therapy journals to capture any articles that were missed in database searches [29, 31, 34].

**Figure 2:**
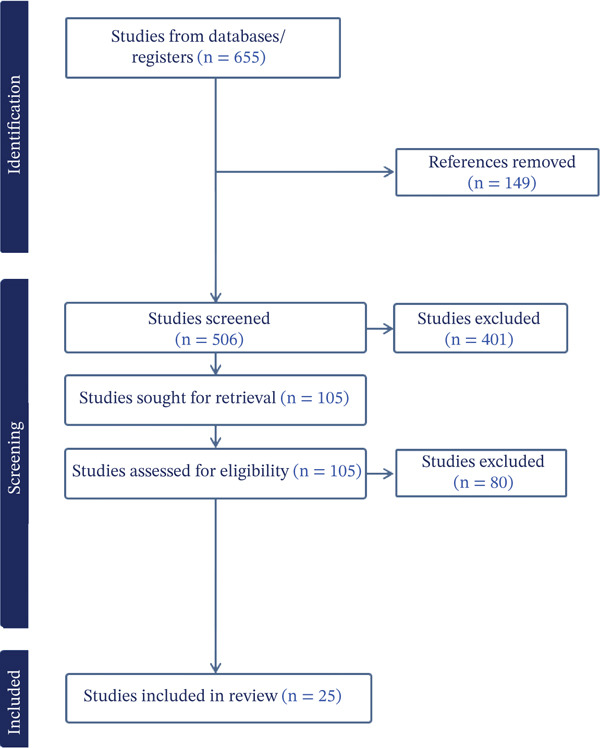
PRISMA diagram.

Search strategy development began with initial presearches in PubMed and EBSCO CINAHL using facets of the PCC format to identify representative articles [29, 31, 32, 34]. An analysis of the text words contained in the title and abstracts of retrieved papers along with index and thesaurus terms was then completed. The research team deduced additional synonymous free‐text words and phrases to articulate the scope of inquiry′s main concepts of *health promotion,* HWC, and *occupational therapy*. An initial search strategy was devised, peer‐reviewed, refined, finalized, and applied, first in Ovid MEDLINE, and then translated for application in EBSCOhost CINAHL, Ovid APA PsycInfo, and PubMed. Date of conducted search was 12/15/2025 with 655 results (See Figure 2).

The Ovid MEDLINE search terms utilized were as follows: “occupational therapist∗”.mp. or exp Occupational Therapists/or exp Occupational Therapy/or exp Allied Health Personnel/or “allied health personnel”.mp. or “physiotherap∗”.mp. AND coaches/or coaching/or coach∗.mp. or “coaching intervention”.mp. or “goal∗”.mp. or exp Goals/or exp Motivational Interviewing/AND exp Health Behavior/or “health behavior”.mp. or Health Promotion/or “health promotion”.mp. or exp Motivation/or “motivation”.mp. or exp Client Centered Therapy/or “client centered care”.mp. or “client centered therapy”.mp. or exp Biopsychosocial Approach/or “biopsychosocial intervention”.mp. or “psychosocial support”.mp. or “Professional‐Patient Relations”.mp. or “patient‐provider interactions”.mp. The complete search strategies for all databases, including all applied filters and limits, are available as an online appendix within the registered protocol at the following DOI: [10.17605/OSF.IO/XPE95].

The articles retrieved from each database were collated and uploaded to Zotero 6.0/2023, and duplicate sets were removed, resulting in 655 studies transferred into Covidence [35] (Covidence, Melbourne, Australia) Software System for further review. Once in Covidence, two authors independently screened title/abstract eligibility per a priori protocol. A third author was available to resolve any disagreements in inclusion. The process was repeated for the subsequent full‐text review stage and all extracted data were entered into a data extraction form for analysis and summarization of results. In the final phase of the article search methodology, the researchers examined selected article reference lists and key occupational therapy journals for any additional eligible articles missed in the database searches including the American Journal of Occupational Therapy and subsequent citations within selected publications. Throughout the project, the reviewers integrated routine and ad‐lib check‐ins to discuss process and progress [29, Blum et al., 2023, 34].

## 3. Results

The original literature search yielded 655 articles with 149 duplicates removed for a total of 506 studies screened by the research team. Of the 506 studies, 401 studies were determined to be irrelevant or ineligible in the title and abstract portion of the selection process. A total of 105 studies were included for full‐text review, and of these 105 articles, 80 were excluded due to not meeting inclusion criteria. A total of 25 articles were included for further examination in this scoping review.

### 3.1. Study Characteristics

Please see Table 1 for a full representation of the included studies. Studies were conducted in the United States (*n* = 14), Canada (*n* = 2), Japan (*n* = 1), Ireland (*n* = 2), Australia (*n* = 2), Sweden (*n* = 1), England (*n* = 1), Netherlands (*n* = 1), and Spain (*n* = 1). Study participant numbers ranged from 2 [52] to 137 [57]. Details on age, gender, and other demographic factors were diverse, with some studies giving detailed participant data and other studies focusing on secondary data collection or qualitative descriptions, thus having little to no participant demographic characteristics provided. The full selection process is detailed in the PRISMA flow diagram (See Figure 2).

**Table 1:** Included articles.

Author(s)/year	Country of origin	Health promotion intervention	Population and sample size	Research design	OT role
[36]	United States	Educational course aimed at improving health literacy and finding health literature online. Aims included improving health management skills through increased self‐efficacy.	Individuals 18 years and older who can make informed decisions regarding their or. *N* = 103	Presurvey/postsurvey design using 4‐point Likert‐scale questions.	Provided in person by an occupational therapist occupational therapy doctoral students with a health sciences librarian.
[37]	Australia	Health Promoting Activity Coaching (HPAC): A 6‐session coaching intervention focused on increasing maternal participation in health‐promoting activities and improving mental health.	Mothers of children with a disability (ages 0–18) who were receiving pediatric OT services. *N* = 16 (Intervention: 11, control: 5)	Nonrandomized controlled pilot feasibility study and specific preoutcome/postoutcome measures.	Pediatric occupational therapist delivered the 10‐min coaching sessions as an add‐on to their standard pediatric therapy sessions.
[38]	United States	Feasibility testing of a Lifestyle Redesign‐inspired program called Aging Well by Design for community‐dwelling older adults.	Community‐dwelling adults aged 65 years or older. *N* = 13	Mixed methods including presurveys/postsurveys, interviews, and specific measures.	Occupational therapists led the program, conducted the research, and designed the implemented protocol.
[39]	Ireland	8‐week program titled “Operation Transformation”. Included exercise and healthy eating classes, and a weight management course.	Individuals participating in a community mental health program. *N* = 41	Mixed methods including postprogram surveys and focus groups.	Occupational therapists implemented and led the program with other health healthcare staff.
[40]	Australia	An intervention was trialed called “Health Promoting Activity Coaching” which stood within the “Healthy Mother Healthy Families Programme”. Aimed at increasing health and wellbeing in mothers of children with disabilities.	Mothers caring for children with a disability. *N* = 11	Qualitative design using semistructured interviews and thematic analysis.	Pediatric occupational therapists provided the intervention after a 4‐h training on health promoting activity coaching.
[41]	United States	Group sessions based on identified meaningful activities. Focuses included physical wellness, healthy leisure participation, and social participation. Goal was to increase perceived health and wellbeing.	University students, staff, or faculty over 18 years of age. *N* = 21	Repeated measures design using surveys and specific measures.	Occupational therapy faculty designed and administered the research intervention and protocol.
[42]	Sweden	A 4‐month occupational‐based health‐promoting program for older adults to maintain and/or improve general health and wellbeing. Additional aims included improving participants′ occupational adaptation.	Community‐dwelling adults aged 65 years or older. *N* = 40	Quasi‐experimental design with a nonequivalent control group with semistructured interviews and presurveys/postsurveys.	Occupational therapists developed and led the research protocol and analysis as well as provided the intervention and program.
[43]	United States	7‐week health occupations program aimed at addressing physical, social, spiritual, among others, components to intervention to improve healthy occupational engagement.	Females between the ages of 11 and 15 years, and members of an identified after school club. *N* = 5	Qualitative phenomenological design. Included preinterviews and postinterviews and observations throughout program implementation.	Occupational therapists designed and implemented the intervention program.
[44]	United States	A program titled the “Healthy Hearts Program” was implemented for 6 weeks with participants and focused on healthy time and weight management and reduction of screen time.	Parent‐child dyads with inclusion criteria for children to be between the ages of 8 and 9 from a community recreation program. *N* = 20 (10 dyads)	Mixed‐methods sequential design with preinterviews/postinterviews for child participants and questionnaires and postprogram interviews for parent participants.	Occupational therapists designed and implemented the intervention program.
[45]	United States	Occupation‐based wellness program that could be individually tailored to support lifestyle change in parents with children with autism spectrum disorder.	Parents who served as primary caregivers for a child on the autism spectrum, 8–21 years of age. *N* = 28	Recursive multistep collaborative process including multiple focus groups, iterations of intervention design and implementation, and qualitative analysis of findings.	An occupational therapist academic faculty and research team designed and implemented the program.
[46]	United States	*Healthy Choices for Me Program* focused on increasing physical activity and healthy foods to increase self‐efficacy in healthy lifestyle choices.	Inclusion criteria for participating schools: in ethnically diverse and low socioeconomic areas in the surrounding city. *N* = 27.	Quasi‐experimental design with one intervention group evaluated pretest and posttest.	Occupational therapists and students were involved in the program′s implementation.
[47]	Canada	The French‐Canadian Lifestyle Redesign Program addressing areas such as healthy aging and health through occupation.	Participants aged 65 or older. *N* = 17	An exploratory descriptive qualitative clinical research design using a convenience sample.	Occupational therapists implemented the program and developed the design of the study.
[48]	United States	Management of wellbeing in caregivers of clients undergoing hematopoietic stem cell transplantation titled CARE (connect, actively relax, exercise).	Caregivers over the age of 18 and providing care for an adult with the condition. *N* = 20	Single‐arm prepost design to assess feasibility of intervention and study procedures.	Occupational therapists implemented the evaluated program in the study.
[49]	Ireland	Fatigue and Activity Management Education (FAME), program for individuals with systemic lupus erythematosus (SLE). Focused on exercise, nutrition, stress management, and fatigue.	Individuals with a definite diagnosis of SLE over the age of 18. *N* = 21	Mixed‐method design including multiple quantitative standardized measures and focus group interviews.	The program was facilitated by an occupational therapist and included multidisciplinary input.
[50]	England	The Fibromyalgia Self‐Management Program (FSMP) focused on goal setting, pacing, sleep hygiene, and nutrition.	Participants with a fibromyalgia diagnosis over 18 years of age. *N* = 74	An exploratory, parallel‐arm, one‐to‐one, randomized controlled trial design was utilized.	Program was delivered by occupational therapists and physiotherapists at two community sites.
[51]	United States	Community program titled “Occupational Therapy Health and Wellness Program (OT‐HAWP) intended to impact perceived performance in daily activities, health‐related quality of life.	Participants with past or present diagnosis of cancer at least 18 years old. *N* = 34	Prospective one group pretest–posttest design to measure effects of the program using multiple standardized measures.	Program was delivered by occupational therapists.
[52]	Netherlands	Healthy and Active Aging Program supported seniors to discover what their daily activities mean for their perceived health and well‐being. A Dutch version of this program was implemented and evaluated.	Community dwelling seniors aged 55 years or older. *N* = 2	Naturalistic single case study, including the use of document analysis, observations, and interviews.	Occupational therapists implemented the new Dutch program and were evaluated based on their experiences in doing so.
[53]	United States	The program “¡Vivir Mi Vida! (Live My Life!)” was evaluated and designed to improve health and well‐being of high‐risk Latino adults receiving rural primary care.	Latino participants aged 50–64‐years old. *N* = 37	Mixed methods design evaluating presurveys–postsurveys and use of a semistructured interview of participants.	The program was delivered by occupational therapists and community health workers.
[54]	United States	Evaluation of the occupation‐focused program “Living Better With What You Have” which includes goals related to reducing food insecurity to improve health.	Participants eligible if they participated in or had graduated from a national Bridges Out of Poverty Getting Ahead program. *N* = 16	Participatory action design with instruments focusing on food insecurity, a food resource profile, and standardized outcome measures.	Occupational therapists conducted the program and were identified as major stakeholders.
[55]	United States	Pilot study of “Powerful Tools for Caregivers” (PTC) telehealth program aimed at improving caregiver wellness by reducing stress, caregiver burden, and improving self‐care, and well‐being.	Active caregivers *N* = 4	Phenomenological approach to understand participant experience. A focus group was conducted following the program.	Occupational therapists composed the research team and designed the study.
[56]	United States	Familias Saludables (healthy families) program aimed at fostering family routines and goals that improve health behaviors among Latino youth with disabilities along with their families.	Families with children with disabilities between ages 8‐ and 22‐years receiving services from a local community agency. *N* = 19	Predesign/postdesign with follow‐up using specific measures.	Occupational therapy students assisted and provided aspects of the program.
[57]	Japan	Program aimed at improving satisfaction with occupation and life satisfaction in community dwelling older adults using an occupational journal.	Participants aged 65 years or older. *N* = 137	Predesign/postdesign with a case control conducted following a randomized clinical trial (prior publication).	Main author, who is an occupational therapist, led the program and all sessions it provided.
[58]	United States	Occupation‐Based Coaching (OBC): A metacognitive strategy training intervention delivered via telehealth (8 sessions) to address activity‐based goals and participation.	Autistic children (ages 5–12) and their primary caregivers. *N* = 13 (13 children and 12 caregivers).	Single‐group pretest–posttest design (pilot feasibility study) with preintervention and postintervention outcome measures.	Occupational therapists served as coaches in the program.
[59]	Spain	Program consisting of group or individual occupational therapy treatment focused on improving independence in daily activities, self‐efficacy, and wellness in older adults.	Inclusion criteria were the ability to read and a normal cognitive function score on the Mini‐Mental Scale. *N* = 74	A randomized preclininal/postclinical trial to assess the effects of group versus individual occupational therapy intervention.	Occupational therapists conducted the intervention and ran the evaluated program.
[60]	Canada	In‐home transfer training skills intervention by occupational therapists. Health‐promoting interventions were also provided including personal care and mobility and evaluating client health literacy.	Inclusion criteria from original study was not included. Occupational therapists evaluated were working in six different Health and Social Service Centers. *N* = 11.	Secondary data analysis of a qualitative study conducted prior to using semistructured interviews and recorded audio tapes. Qualitative coding of data and descriptive statistics were run.	Occupational therapists provided the original intervention in the study data being evaluated.

### 3.2. Interventions

A diverse array of interventions was highlighted in the studies included. These included programs specifically focused on improving health and well‐being or health‐promoting topics such as exercise and weight management [39, 41, 44, 46], whereas others analyzed health and wellness management outcomes as additional or secondary to the primary intervention focus [36, 38]. Other studies such as Polo and Sommers (2022) focused on improving health and wellness specifically through occupational engagement such as social and leisure activities [43, 57]. Additionally, coaching‐based interventions emerged as a significant trend, focusing on health‐promoting activity coaching for maternal mental health [37] and occupation‐based coaching (OBC) for autistic children [58]. One study focused specifically on the topic of health coaching with an array of healthcare professionals including occupational therapists [40]. Many of the programs highlighted in the included manuscripts focused on a specific health condition or diagnosis [48, 49, 50, 58] whereas others, such as [Cassidy et al. 38] were generalized to a specific age group or population such as community‐dwelling older adults [42, 57, 59] or caregivers [37, 45] See Table 2 for a full list of interventions utilized in these studies.

**Table 2:** Interventions in included studies.

Health and wellness coaching skills highlighted in included studies based on operational definition by Sforzo et al. [10] and National Board Certification Definition from NBC‐HWC	
**Interventions addressing multiple components of health (physical, social, spiritual, mental, etc.) (** **n** = 19**)**	[37]; [39]; [40]; [41]; Janssen et al. 2021; [42]; Kim et al. 2022; [43]; [45]; [46]; [48]; [49]; [50]; [51]; [52]; [53]; [55]; [56]; [58]
**Collaboration with other professions or support systems to implement a program or topic (** **n** = 5**)**	[36]; Booth and Nelson 2013; Davies et al. 2020; Janssen et al. 2021; [53]
**Topic or intervention included goal setting component (** **n** = 16**)**	[37]; [38]; Davies et al. 2020; [40]; [41]; [42]; [43]; [45]; [46]; [47]; [48]; [51]; [53]; [54]; [56]; [58])
**Focus on self-efficacy or perceived health in participants (** **n** = 7**)**	([36]; [37]; [42]; [46]; [48]; [50]; [59]
**Behavior-focused interventions or topics (** **n** = 14**)**	[37]; [38]; [39]; [40]; [42]; J. [44]; [45]; [46]; [48]; [49]; [50]; [53]; [56]; [58]
**Focus on skill-building to increase healthy behaviors or outcomes (** **n** = 10**)**	([36]; Davies et al. 2020; J. [44]; [43];[49]; [50]; [51]; [54]; [58]; [59])
**Notes using a working relationship/partnership with client (** **n** = 4**)**	(Booth and Nelson 2013; [37]; [52]; [58])
*Health promotion topics and interventions highlighted in included studies*	
**Improving health literacy (** **n** = 2**)**	([36]; [60])
**Health Promotion related to a specific diagnosis (** **n** = 5**)**	(Booth and Nelson 2013; [49]; [50]; [51]; [58])
**Health and wellness promotion through tailored occupational therapy, or related intervention (** **n** = 12**)**	([37]; [38]; Davies et al. 2020; [40]; Janssen et al. 2021; [44]; [43][47]; [48]; [52]; [53]; [56]; [58])
**Program focuses on healthy eating, sleep, exercise participation, leisure, weight management, or related topic to improve health and wellness (** **n** = 20**)**	([37]; [39]; [40]; [41]; Janssen et al. 2021; [42]; [44]; [44]; [45]; [46]; [47]; [48]; [49]; [50]; [51]; [52]; [53]; [56]; [58]; [60])
*Studies using occupation-based health and wellness interventions*	
**Occupation-based interventions (** **n** = 11**)**	([38]; [41]; [42]; [44]; [45]; [47]; [51]; [52]; [57]; [58]; [59])

### 3.3. Measurement and Instruments

There was a wide variety of assessment tools utilized to assess the effectiveness of these health and wellness programs. The assessments cited the most were the Canadian Occupational Performance Measure (COPM) (*n* = 4) [43, 51, 54, 58], the Meaningful Activity Participation Assessment (*n* = 3) [38, 41]; A. [42]) and the SF‐36 (*n* = 3) [38; A. [42, 50], and the Depression Anxiety Stress Scale (DASS‐21) [37]. See Table 3 for a detailed list of all 57 assessments reported in our findings.

**Table 3:** Outcome measures in included studies.

Outcome measures reported in included studies (*n* = 57)			
18‐Question survey about family rules and routines	[56]	Jenkins Sleep Scale (JSS)	[53]
ADLSS	[57]	K–I Scale for the Feeling that Life is Worth Living among the Aged	[57]
American Occupational Therapy Association Occupational Profile Template	[41]	Kiresuk and Sherman’s (1968) Goal Attainment Scaling	[56]; [58]
Arthritis Self‐Efficacy Scale‐8 (ASES‐8)	[50]	Short Report Measure of Life Satisfaction (LSI‐Z)	[57]
Beach Center Family Quality of Life Scale	[58]	Making Meals Performance Measure	[54]
Block 2005 Food Frequency Questionnaire Spanish Version	(Schepens [53])	Meaningful Activity Participation Assessment	[38]; [41]; [42]
Brief COPE	[48]	Meaningful and Psychologically Rewarding Occupation Rating Scale	[41]
Canadian Occupational Performance Measure (COPM)	[43]; [51]; [54]; [58])	Measure Yourself Medical Outcome Profile 2 (MYMOP2)	[53]
Caregiver Self‐Efficacy Questionnaire	[48]	Multidimensional Assessment of Fatigue (MAF)	[51]
CATCH Kids Club Questionnaire	[43]; [46])	Parenting Sense of Competence Scale	[58]
Center for Epidemiologic Studies Depression Scale	70	Patient Activation Measure 12‐item Short Form (PAM‐13)	[53]
Chalder Fatigue Scale (CFS) Questionnaire	[50]	PROMIS‐Global Health	[51]
Child and Adolescent Scale of Participation	[58]	Perceived Stress Scale	[45]
Child and Adolescent Trial for Cardiovascular Health: Health Behavior Questionnaires (HBQ)	[46]	Physical Activity Questionnaire (elementary school)	[46]
Depression, Anxiety, and Stress Scale—21 Items	[37]	Pittsburgh Sleep Quality Index (PSQI)	[51]; [53]
Distress Management Questionnaire (NCCN)	[48]	Pizzi Healthy Weight Management Assessment	[44]
Energy Conservation Strategies Survey	[49]	PROMIS Pediatric Sleep Measures	[58]
Engagement in Meaningful Activities Survey	[38]	RAND 36‐Item Short Form Survey	[41]
EQ‐5D‐5L	[50]	Revised Fibromyalgia Impact Questionnaire (FIQR)	[50]
Fatigue Severity Scale	[49]	Ryff’s Psychological Well‐Being Scale	[59]
Frenchay Activities Index (FAI)	[49]	Self‐Efficacy for Performing Energy Conservation Strategies Assessment (SEPECSA)	[49]
Functional Assessment of Cancer Therapy‐Bone Marrow Transplant FACT‐BMT	[48]	SF‐36	[38]; [42]; [50])
General Self Efficacy Scale (GSE)	[59]	Symptom Burden Questionnaire	[48]
Geriatric Depression Scale–Short Form	([38]; [59])	The Barthel Index	[59]
Goal‐setting form adapted from the Health Matters curriculum by Marks et al. (2010)	[56]	The Food Security Scale	[54]
Health Promoting Activities Scale	[37]	The Individual Food Resource Profile (IFRP)	[54]
Health Education Impact Questionnaire (HEIQ)	[49]	The Lupus Quality of Life Questionnaire	[49]
Hospital Anxiety and Depression Scale (HADS)	[49]	Warwick‐Edinburgh Mental Well‐Being Scale	[41]
International Physical Activity Questionnaire (IPAQ)	[53]		

## 4. Discussion

This scoping review is aimed at exploring the interplay between occupational therapy and HWC strategies within health‐promoting interventions. The findings suggest that occupational therapists are uniquely positioned to utilize formalized HWC approaches, given the established efficacy of HWC component parts found across the included studies. The evidence illustrates an integral role for occupational therapy in health promotion through the application of specific HWC skillsets, notably goal setting, multimodal holistic foci, and client‐centered methodologies (see Figure 1 for the conceptual overlap between professions). This connection is further underscored by the observed links between occupation‐based wellness interventions and improved participant health outcomes. Although a formal integration of HWC within occupational therapy is not yet standardized, these results demonstrate a significant existing synergy between the two fields. Despite the diversity of the included studies, a consistent theme emerged: Occupational therapists effectively deliver health‐promoting interventions using skillsets that parallel those of HWC. Recent research by Bourke‐Taylor et al. [37] and Tkach et al. [58] further illustrates this movement toward formalization, utilizing protocols such as Health Promoting Activity Coaching (HPAC) and OBC to bridge the gap between traditional occupational therapy and structured wellness coaching.

Within the literature, nursing is frequently cited for its use of HWC coaching techniques across various nursing settings [61, 62, 63]. In contrast to the broad occupational focus found in this review, many cited instances of HWC in other professions tend to focus on specific health behaviors, such as smoking cessation or tobacco usage [64, 65]. Our scoping review differentiates itself from existing literature by using an operational definition of HWC [10] and highlighting the striking similarity in skillsets between HWC and the field of occupational therapy. This commonality is evident in the profession′s overarching framework and the application of skillsets in intervention and practice. While these similarities were identified, there is not a clearly defined framework within the field of occupational therapy to guide health promotion‐specific interventions. This review assists in introducing an intervention that is both novel and established in its effectiveness, offering a potential framework to support the occupational therapy profession in this domain of practice. This is exemplified by Table 3, which shows a heterogenous mix of assessments used to measure patient health and well‐being, illustrating the lack of consistency in how occupational therapy practitioners treat and assess health and well‐being in clinical populations. Through a more formalized health promotion intervention approach, such as HWC, there is room to streamline effective outcome assessments utilized in this subset of the field.

The occupational therapy profession has a longstanding tradition of prioritizing the holistic well‐being of clients, encompassing the physical, mental, and social dimensions of health. The realm of health promotion is embedded in the field, with literature regarding the need for and implementation of health‐promoting interventions on the rise; the occupational therapy profession can help fill a growing need [1; Early et al. 2019; 66]. HWC has, in tandem, been established as a health promotion intervention as exemplified by studies spanning multiple health professions. A study by Cinar et al. [67] illustrated HWC as a health promotion intervention that was more effective than health education alone in clients with diabetes. Another study by Gefter [68] demonstrated comparable results using HWC as a health promotion program for urban and rural youth. Our findings underscore that health promotion is not a recent addition to occupational therapy practice but an integral and foundational component. Yet, there is room for increasing consistency in our approach to addressing it based on the diverse findings of our review.

A second theme that emerged from our scoping review is the intrinsic relationship between occupational performance and healthy behavior engagement. This provides the unique edge of occupational therapy practice and its use of health‐promoting interventions from other healthcare professions. Clients who can effectively participate in meaningful activities, occupations, and routines are more likely to adopt and sustain healthy lifestyle changes [69]. This insight stresses the unique potential of occupational therapists to collaborate with clients in identifying and addressing barriers to healthy behaviors, contributing to improved overall well‐being. Again, in parallel, HWC aims to do the very same thing by helping clients navigate barriers to healthy behavior change [22]. By recognizing the role of occupational performance as a catalyst for healthy behavior change, occupational therapists may be well‐positioned to integrate HWC as a formal component of holistic practice, complementing their existing role in health promotion.

It is important to highlight these patterns that emerged from this review to acknowledge the breadth of occupational therapy practice in health promotion and where it can continue to improve. This review spotlights the similarities of HWC skill sets used in occupational therapy interventions toward health promotion; it also underscores how the success of many of these interventions was using occupation‐based treatment, which demonstrates striking similarities to the process of HWC. Yet, there was such diversity and heterogeneity in the measures used and interventions implemented in these studies that we believe it is apparent that there is a need within the occupational therapy profession for a formalized approach to health promotion when treating clients. HWC, based on its emergence in the literature, used alongside occupation‐based treatment to promote health, may provide a more structured intervention modality that can promote health without losing its individualistic and holistic approach foundational to occupational therapy practice.

While our scoping review emphasizes the similarities between health coaching and occupational therapy, it is essential to recognize that occupational therapy has historically encompassed a wide range of practices that extend beyond coaching. Occupational therapy interventions often focus on physical rehabilitation, adaptive equipment training, and environmental modifications, per the Occupational Therapy Practice Framework: Domain and Process [70], which are distinct from the personalized, goal‐oriented approach of coaching [12]. Although occupational therapy has traditionally navigated the tensions between the medical model and client‐centered practice, HWC offers a specialized, goal‐oriented framework that can further augment the profession′s collaborative, self‐discovery–based interventions. [71]. By integrating coaching methodologies, occupational therapy can enhance its practice by adopting innovative strategies that complement traditional interventions, thereby enriching the therapeutic process and offering new perspectives.

## 5. Limitations of the Scoping Review

Despite the evident synergy between occupational therapy and HWC, we encountered a significant challenge during our scoping review: the limited availability of comprehensive descriptions of HWC practices within the context of occupational therapy. This dearth of detailed information poses a barrier to practitioners seeking to incorporate HWC principles into their therapeutic approaches. To address this issue, it is essential to prioritize the development of standardized frameworks and guidelines specific to occupational therapy. These guidelines should offer practical insights into the seamless integration of HWC skills into occupational therapy practice, bridging the existing knowledge gap and providing occupational therapists with an evidence‐based skill set to formally address health promotion needs of every client. Additionally, the wide array of assessments used in the included studies demonstrates a lack of established outcome measures used in occupational therapy health‐promoting interventions, perpetuating a call for further research in identifying and/or developing these tools.

## 6. Future Research Directions

As occupational therapy embraces new intervention styles, modalities, and settings, the opportunity for the integration of HWC practices in the field grows. Now that evidence‐based educational standards for HWC exist and a national board certification has been developed, studies highlighting the formal usage of HWC can be evaluated in the field of occupational therapy. Further research of formal integrative HWC in occupational therapy is needed to further analyze its efficacy as an embedded practice in the field.

The diversity and complexity of assessments used within the included studies led to confusion regarding how health and health promotion are measured and evaluated. Fifty‐seven assessments were used in the included studies with little repetition of use among authors. As a profession, there is an urgent need to delineate clear and standardized measures for health‐related outcomes, including the assessment of health behaviors and the impact of occupational therapy interventions on health promotion. This clarity is essential for demonstrating the contributions of occupational therapists to health promotion and well‐being while ensuring consistency in practice.

## 7. Conclusion

In conclusion, our scoping review contributes valuable insights into the convergence of occupational therapy and HWC, emphasizing the shared principles and potential for the melding of these domains. The integration of HWC concepts into occupational therapy practice holds substantial promise for enhancing client outcomes and solidifying the profession′s role in the management of lifestyle disease through health promotion. As we move forward, addressing the challenges, developing clear frameworks, and conducting further research are critical steps in cementing the integration of HWC within occupational therapy practice. This integration can empower clients to lead healthier lives, aligning with the core values and mission of the occupational therapy profession.

## Author Contributions

Alyssa M. Smith: conceptualization (equal), data curation, formal analysis, investigation (equal), methodology (equal), writing—original draft (equal), writing—review and editing (equal). Kelli Kauffroath: conceptualization (equal), investigation (equal), methodology (equal), writing—original draft (equal). Karen Westervelt: conceptualization (equal), methodology (equal), Writing —review and editing (equal). Victoria Priganc: conceptualization (equal), methodology (equal), writing—review and editing (equal).

## Funding

No funding was received for this manuscript.

## Disclosure

All authors reviewed, edited, and approved the final version of the manuscript and agreed to take responsibility for all aspects of the work.

## Ethics Statement

This scoping review follows the PRISMA‐ScR reporting standards. The research involves solely secondary analysis of published literature and is classified as nonhuman subjects research under the Declaration of Helsinki and U.S. Forty‐five CFR 46; therefore, formal institutional ethical oversight was not required.

## Conflicts of Interest

The authors declare no conflicts of interest.

## Data Availability

Data is available upon request from the authors.
